# Monitoring Menstrual Health Knowledge: Awareness of Menstruation at Menarche as an Indicator

**DOI:** 10.3389/fgwh.2022.832549

**Published:** 2022-03-24

**Authors:** Julie Hennegan, Zay Yar Swe, Kyu Kyu Than, Calum Smith, Lidwien Sol, Hilda Alberda, Justine N. Bukenya, Simon P. S. Kibira, Fredrick E. Makumbi, Kellogg J. Schwab, Peter S. Azzopardi

**Affiliations:** ^1^Global Adolescent Health Group, Maternal Child and Adolescent Health Program, Burnet Institute, Melbourne, VIC, Australia; ^2^Melbourne School of Population and Global Health, University of Melbourne, Melbourne, VIC, Australia; ^3^Myanmar Country Program, International Development Discipline, Burnet Institute, Yangon, Myanmar; ^4^Irise International, Sheffield, United Kingdom; ^5^School of Business and Economics, Maastricht University, Maastricht, Netherlands; ^6^Simavi, Amsterdam, Netherlands; ^7^Department of Community Health and Behavioural Sciences, School of Public Health, College of Health Sciences, Makerere University, Kampala, Uganda; ^8^Department of Epidemiology and Biostatistics, School of Public Health, College of Health Sciences, Makerere University, Kampala, Uganda; ^9^Department of Environmental Health and Engineering, Johns Hopkins Bloomberg School of Public Health, Baltimore, MD, United States; ^10^Adolescent Health and Wellbeing Program, Aboriginal Health Equity Theme, South Australian Health and Medical Research Institute, Adelaide, SA, Australia

**Keywords:** menstrual health, menstrual literacy, menstrual hygiene, adolescent girls, survey, adolescent health, wellbeing

## Abstract

As initiatives to support menstrual health are implemented globally, monitoring progress through a set of comprehensive indicators provides important feedback to direct policies and programs. One proposed core indicator is awareness of menstruation at menarche. That is, at the time of menarche an adolescent girl knowing that menstrual bleeding is something she will experience. In this investigation, we undertook secondary analysis of data collected across four studies to support interpretation of this indicator. We (1) describe the proportion of each sample aware of menstruation at menarche, (2) test variations in awareness according to sociodemographic characteristics, and (3) describe the associations between this indicator and self-reported experience at menarche, social support, and confidence to manage menstruation. Studies included cross-sectional survey data from 421 schoolgirls in Magway, Myanmar, 537 schoolgirls in Soroti, Uganda, 1,359 schoolgirls in Netrokona, Bangladesh, and 599 adult women working in Mukono, Uganda. Awareness of menstruation at menarche varied from 84% in Myanmar to 34% in Bangladesh. Older age at menarche was associated with awareness. Awareness at menarche was not associated with household poverty in the adolescent samples, but greater poverty was associated with lower levels of awareness among adult women. In Myanmar, girls aware of menstruation had significantly higher odds of reporting that they felt prepared (2.85 95% CI 1.34–6.08), happy (OR = 3.81 95% CI 1.74–8.37) and knew what was happening at menarche (OR = 2.37 95% CI 1.34–4.19). However, they also reported higher levels of embarrassment (OR = 1.76 95% CI 1.04–2.97) and did not report significantly less fear (OR = 1.24 95% CI 0.82–1.85). Awareness of menstruation at menarche was associated with higher scores on a menstrual knowledge quiz in both Myanmar (b = 9.51 95% CI 3.99–15.04) and Bangladesh (b = 4.78 95% CI 1.70–7.87). In these studies girls aware of menstruation at menarche also had higher odds of reporting they felt confident discussing menstruation with support sources and managing menstruation at school, while these differences were not significant among schoolgirls in Uganda. Findings support the usefulness of awareness of menstruation at menarche as an indicator to describe minimal knowledge of menstruation and suggest that awareness may signal greater knowledge, social support, and confidence in some settings.

## Introduction

Menarche, the first menstrual period, is an important pubertal milestone in adolescence. It marks a biological, and often social transition, and the onset of accompanying menstrual health needs (1). Menstrual health, a state of physical, mental and social-wellbeing in relation to the menstrual cycle, requires timely knowledge about the menstrual cycle, access to resources and facilities to care for the body, support for discomforts and disorders, and a supportive socio-cultural environment free from exclusions and restrictions related to menstruation (2). There is increased recognition that menstrual health is essential for human rights (3, 4), gender equality (5–7), and achieving the Sustainable Development Goals (8).

Awareness of menstruation prior to menarche has been proposed as a core indicator for monitoring progress toward menstrual health at national and global levels. The indicator was suggested following global expert consultation for monitoring menstrual health and hygiene (9) and incorporated into UNICEF's Guidance for Monitoring Menstrual Health and Hygiene (10). It has been highlighted by the Joint Monitoring Programme (JMP) of the World Health Organisation and United Nations Children's Fund as a priority domain for national monitoring in their most recent progress update (11). It has also been proposed as an indicator for monitoring global adolescent health by the Global Action for Measurement Of Adolescent Health Advisory Group (12, 13). Despite recommendations from these groups, the indicator is yet to see significant uptake and there is limited data currently available.

Being aware of menstruation at menarche- that is, an adolescent girl knowing that menstrual bleeding/discharge is something that she can expect to experience- represents the most basic level of knowledge about menstruation. It does not indicate that an adolescent has received sufficient information or capture broader menstrual health literacy, that is, having a sufficient level of knowledge and skills to understand anatomical and biological facts about the menstrual cycle and enact self-care (14). However, it suggests that some minimal information has been provided in time. A large body of qualitative research has consistently found that participants felt underprepared, and report experiencing high levels of fear and distress at menarche if they are unaware of menstruation (15–21). Indeed, this was reported as the most consistent finding across 76 included studies in a systematic review of qualitative research on menstrual experiences in low-and middle-income countries (15). Monitoring awareness at menarche will track the proportion of the population vulnerable to this distressing formative experience. As only a small selection of indicators can be feasibly included in large scale monitoring, awareness of menstruation at menarche may also be used to suggest that an individual has at least one source of information about menstruation. This data may also suggest that school-based menstrual education is being provided in time for menarche. Additional data on the age at menarche in each country may also be needed to target education programs, and has been highlighted as a gap in adolescent health data (1, 22).

Quantitative studies of menstrual health experiences have reported on awareness at menarche to provide an indication of sample menstrual knowledge (23). In a systematic review with searches in 2015, Chandra-Mouli and Patel identified 23 studies from low-and-middle-income countries reporting on awareness of menstruation at menarche, most (*n* = 16) from India (23). Across these studies awareness ranged from 2.8% of girls in Rajasthan, India to 100% among girls in urban Turkey. In 2015, Tamiru et al. reported that in survey data collected in project areas in Ethiopia, South Sudan, Uganda, Tanzania and Zimbabwe, an average 66% of adolescent girls reported not knowing about menstruation at menarche, although did not report a break-down across countries (24). In a survey of post-menarche schoolgirls aged 11–17 across Bangladesh in 2013, 36% reported having knowledge of menstruation before menarche (25).

While studies have used awareness at menarche for descriptive purposes, few have tested relationships with broader menstrual health literacy, experience of menarche, or social support. We may hypothesize that girls aware of menstruation at menarche have greater levels of other menstrual knowledge than their peers. As noted above, this may also indicate that those being told of menstruation have a source of information, and possibly support. Qualitative studies suggest that awareness at menarche may reduce fear and distress. It is not clear if a more positive menarche experience helps to build confidence in managing menstruation or bodily autonomy, although this has been hypothesized (26). In 2021, the JMP has reported that only two countries had national data on awareness of menstruation at menarche (11). Egypt's Survey of Young People in 2014 found that 66% of respondents knew about menstruation before their first period, and in the Bangladesh National Hygiene Survey (participant ages 15–49) only 32% of respondents reported knowing or hearing anything about menstruation before their first period. In Egypt, investigators found that awareness of menstruation prior to menarche was higher among urban than rural participants (27). The JMP analysis reported that there was no difference by rural residence in Bangladesh, but in both countries earlier age at menarche was associated with poorer awareness (11). This analysis also reported greater fear and distress among those who were not aware of menstruation at menarche, although found no relationship with knowing what to do at menarche, and in Bangladesh a greater proportion of girls who were aware of menstruation felt ashamed at menarche (11).

Through the present study we aimed to provide insights on the performance of awareness of menstruation at menarche as an indicator capturing support for menstrual health and inform the interpretation of data as it becomes available. We undertook secondary analysis of survey data from four studies. We (1) report the proportion of respondents with awareness of menstruation prior to menarche in each population, (2) test the variability in awareness according to sociodemographic characteristics and age at menarche, and (3) test the relationships between awareness at menarche and more comprehensive measures of menstrual knowledge, experience at menarche, social support, and confidence to manage menstruation.

## Methods

### Study Populations and Data Collection

In this study we conducted secondary analysis of data collected in four different studies of menstrual experiences. Each study was undertaken for a different primary purpose. Included studies were those in which the first author had been involved and had access to the primary data for exploratory analyses, and that had collected data on knowledge of menstruation at menarche as part of participant surveys. Below we report each study's primary aim, population, sampling strategy and data collection methods. Further details are reported in the cited publications pertaining to each research effort. Our investigation is reported in accordance with the STROBE statement (28). As the included studies were not collected with the aim of addressing our research question, a-priori sample size calculations were not possible.

#### Magway, Myanmar

The first included study was a formative research effort to understand the sexual and reproductive health needs of adolescents to inform a life-skills education program (29). Data were collected in 2016. All Monastic schools in the Magway Region in central Myanmar were invited to participate (*n* = 27), with data collected in 16 rural and semi-rural schools due to heavy flooding. Students aged 11–18 years attending the school on the day of data collection were invited to participate, with 1,427 students (765 girls and 662 boys) completing the self-administered survey. Surveys examined reproductive health knowledge, including 11 questions on knowledge related to menstrual biology. A total of 421 adolescent girls' post-menarche (mean age = 14.0) participated and were asked questions about their menstrual management, experiences of menarche and school attendance related to menstruation.

#### Soroti, Uganda

The second study included was a cross-sectional survey of schoolgirls across 12 schools in Soroti, Uganda undertaken as part of the development and validation of the Menstrual Practice Needs Scale (30) and Menstrual Practices Questionnaire (31). Schools were those engaged with Irise Institute East Africa, a menstrual-health focused non-governmental organization, and were government schools selected by the District Education Office for support. Girls attending school on the day of data collection aged 12 years and older from class levels Primary 5 and Primary 6, and extending to Primary 4 and Primary 5 in some schools (max age 19 years), were invited to participate. Participants self-completed the survey with verbal translation and guidance provided by a research assistant to groups of no more than six. A total of 538 post-menarche girls participated and 537 responded to the question capturing awareness at menarche (mean age = 14.5). Data were collected in 2019.

#### Netrokona, Bangladesh

Our third included dataset was baseline data collected as part of the Ritu clustered randomized controlled trial of a multi-component menstrual health intervention in Netrokona, Bangladesh (32–34). These data were collected in 2017. All junior secondary schools in the district were screened for inclusion, and 149 with no other ongoing NGO programming consented to participate. Schoolgirls in the 6th and 7th grade were eligible, and 28 girls were randomly selected per school for the baseline survey by drawing marbles from a bag. Female enumerators verbally administered the survey and entered participant responses on Android tablets. The survey included questions capturing sociodemographic, gender attitudes, school engagement, menstrual management behaviors, menstrual knowledge and experiences, and school engagement. A total of 1,359 girls post-menarche participated (ages 10–16, mean = 11.8).

#### Mukono, Uganda

The final included study was a cross-sectional survey of adult women (aged 18–45) working in markets, government primary day schools, and government health care facilities in Mukono district, Uganda (35, 36). This study was undertaken to describe the menstrual and sanitation experiences of working women and investigate the impacts on their work and wellbeing. Data were collected through an enumerator-administered survey loaded on smartphones. All markets in the district operating at least 8 h a day and 3 days a week were included, with participants selected systematically. Five teachers and five health care facility workers at services in closest geographic proximity to each market were sampled. A total of 500 women working in markets, 50 teachers, and 50 health care facility workers participated and reported on their awareness of menstruation at menarche. Of this sample, one participant reported not recalling if they were aware of menstruation at menarche and was excluded from analysis. A total of 435 women working in markets, 45 teachers, and 45 health care facility workers had menstruated in the past 6 months and reported on their experience of menstruation (*n* = 525, mean age = 30.6).

### Measures

#### Awareness at Menarche

In each included study, the question used to assess awareness of menstruation at menarche is reported in Table 1.

**Table 1:** Survey questions used to assess knowledge of menstruation at menarche.

Magway, Myanmar	Had you heard about menstruation before you got your first period? Responses: Yes/ No
Soroti, Uganda	When you had your first period, did you know what it was? Responses: Yes/No
Netrokona, Bangladesh	When you had your first MP (menstrual period), did you know what it was? Responses: Yes/No
Mukono, Uganda	Before you had your first menstrual period, were you aware of menstruation? Responses: Yes/No/Don't know

#### Household Wealth and Adult Women's Education Level

Awareness at menarche was compared according to sociodemographic characteristics: poverty and educational attainment for adult women. The most appropriate variable representing household wealth was selected from each study. Both studies undertaken in Uganda used the Lived Poverty Index (37), which asked participants to report how often over the past year participants' household went without resources on a 5-point scale from never to always. For adolescent girls in Soroti girls reported on going without food, water, medicine and school supplies and a mean score (0–4) was calculated with higher scores representing greater poverty. Adult women working in Mukono reported going without food, water, medical treatment, fuel for cooking, and cash income, with a total score (0–20) used for analysis. Notably, this assessed women's current lived poverty. For adult women we also assessed retrospective reports of awareness at menarche according to the highest level of schooling attended.

In Netrokona, Bangladesh, girls reported household resources using the Bangladeshi Simple Poverty Scorecard (PPI) (38) with questions focused on items owned by the household. Possible scores ranged from 0 to 93.

No household resource or poverty index was used in the study in Magway, Myanmar. The study focused on students in the Monastic schools. In these schools, novice nuns and monks are those from poorer households (29), with housing and education costs supported by community donations. Enrolment as a novice nun was thus used to indicate participants from poorer households.

#### Age at Menarche

All three studies of adolescent girls (Magway, Soroti, Netrokona) assessed age at menarche. In Magway and Netrokona, participants were asked to report their age when they had their first period. In Soroti, participants reported if their first period was “this school year,” “last school year,” or “more than two years ago.” Either 0, 1 or 2 years were deducted from participants' self-reported age to estimate age at menarche.

In Mukono, Uganda, adult women were not asked to report their age at menarche but reported their age in years.

#### Experience at Menarche

Only the study undertaken in Magway, Myanmar collected data on girls' experiences at menarche. Participants were asked to think back to their first period and report if they felt: happy, afraid, embarrassed, normal, proud, knew what was happening, prepared, ashamed, knew what to do, was celebrated by family or community, felt comfortable to talk to their family, felt comfortable to talk to female friends, and felt comfortable to talk to male friends. Only six participants felt comfortable to discuss menstruation with males and this item was excluded from comparative analysis. Participants reported if they “*agreed”* that they felt this way, “*disagreed”* or “*did not know”*. For analysis, participants “*agreeing”* with the statement were compared to those who “*disagreed”* or reported that they “*did not know”*.

#### Menstrual Knowledge

Studies in Magway, Myanmar and Netrokona, Bangladesh both included a test to assess participants' knowledge about menstruation and the menstrual cycle. This was 11 items long in Myanmar and five items in Bangladesh. Correct answers were scored and converted to a percentage score for analysis. Studies in Uganda did not include measures of menstrual knowledge.

#### Social Support

All four studies included questions which asked participants to report their confidence or comfort discussing menstruation with support sources, for example in Magway, Myanmar participants were asked if they “agreed” or “disagreed” with the statement “*I feel confident talking about menstruation with a female relative”*. For this investigation, responses across all four studies were dichotomized to represent feeling confident or not feeling confident to discuss menstruation.

#### Confidence to Manage Menstruation

All four studies also asked participants if they felt confident to manage their menstrual bleeding at home or at school. Responses were dichotomized to report if participants felt confident to manage their menstruation at school or work, and if they felt very confident to manage menstruation at home, to maintain consistency with previous studies using these data sets (30, 33).

### Ethical Approvals

Each study received ethical approval consistent with its location and study team:

#### Magway, Myanmar

The study was approved by the Department of Medical Research Ethics Review Committee (035/16) in Myanmar and the Alfred Human Ethics Committee (59/16) in Australia. All participants provided written assent to participate and had written parental consent for participation.

#### Soroti, Uganda

Ethical approval was provided by Johns Hopkins School of Public Health Institutional Review Board (IRB approval no: 00009073) and the Mildmay Uganda Research Ethics Committee (MUREC) (approval ref: 0212–2018). The Uganda National Council for Science and Technology (UNCST) approved the study (ref: SS279ES). Girls provided written informed assent or consent to participate. Parents were informed about the study through information sheets, parent-teacher meetings at each school and through contact with teachers.

#### Netrokona, Bangladesh

This study was approved by the Erasmus Research Institute of Management (IRB 2016-09), Erasmus University Rotterdam, and the Directorate of General Secondary Education in Bangladesh. The District Education Officer also granted approval for data collection and girls provided written informed assent.

#### Mukono, Uganda

Ethical approval was provided by Makerere University School of Public Health Higher Degrees, Research and Ethics Committee (HDREC: 739) and Johns Hopkins Bloomberg School of Public Health Institutional Review Board (IRB: 00010015). The Uganda National Council for Science and Technology (UNCST) approved the study (ref: SS 5143). All women provided written informed consent.

Each study research team provided approval for secondary analysis for this investigation, consistent with existing ethical approvals for exploratory investigations of girls' experience of menstruation and measures of menstrual experience.

### Analysis

Analysis was conducted using Stata 17. For each study, we used descriptive statistics to report the proportion of the sample aware of menstruation at menarche. Bivariate relationships between sociodemographic characteristics (poverty, and age and schooling for adult women), age at menarche, and awareness were tested using binary logistic regression with adjustment for clustering at the school (or workplace) level. In study data from Myanmar, we compared girls' reported experiences at menarche according to their awareness of menstruation, presenting descriptive statistics and binary logistic regression with cluster adjustment.

Menstrual knowledge test scores were a continuous outcome variable. Their association with awareness at menarche was tested using ordinary least squares regression with adjustment for clustering within schools. Social support, and confidence to manage menstruation were compared according to awareness status using binary logistic regression with cluster adjustment for the three studies among adolescent participants. We specified a-priori that if any relationship between sociodemographic characteristics (poverty, adult women's age and education level) and awareness at menarche (*p* < 0.10) were identified, these would be adjusted for in these comparisons as potential confounders. This was only the case for adult women in Mukono, Uganda, and so multivariable logistic regressions with adjustment for clustering and household resources were undertaken for this study.

## Results

### Awareness at Menarche

Awareness of menstruation at menarche varied widely between the study samples. In Magway, Myanmar 84% of schoolgirls knew about menstruation prior to menarche, among schoolgirls in Soroti, Uganda this was 45%. Awareness was lowest in Netrokona, Bangladesh, with 34%. Among adult women in Mukono, Uganda half (49%) reported having been aware of menstruation at menarche.

Awareness at menarche according to age at menarche and sociodemographic characteristics are displayed in Table 2. In all three adolescent samples, increasing age at menarche was associated with increased awareness although this difference was not statistically significant in the sample in Soroti, Uganda.

**Table 2:** Awareness of menstruation at menarche, according to household wealth, age at menarche, and age.

**Awareness at menarche**	**Myanmar (*****n*** **=** **421)**			**Uganda, Soroti (*****n*** **=** **537)**			**Bangladesh (*****n*** **=** **1,359)**			**Uganda, Mukono (*****n*** **=** **599)**		
	**Yes% (*n*)**	**No% (*n*)**	**OR (95%CI)**	**Yes% (*n*)**	**No% (*n*)**	**OR (95%CI)**	**Yes% (*n*)**	**No% (*n*)**	**OR (95%CI)**	**Yes% (*n*)**	**No% (*n*)**	**OR (95%CI)**
	84.1 (354)	15.9 (67)		44.7 (240)	55.3 (297)		34.4 (468)	65.6 (891)		49.3 (296)	50.5 (303)	
**Age at menarche**												
Continuous			1.56 (1.19–2.06)			1.19 (0.98–1.44)			1.31 (1.13–1.52)			
11 & younger	83.3 (25)	16.7 (5)		31.6 (6)	68.4 (13)		31.8 (285)	68.2 (611)				
12	75.0 (96)	25.0 (32)		47.3 (35)	52.7 (39)		39.8 (162)	60.2 (245)				
13	85.8 (145)	14.2 (24)		38.0 (68)	62.0 (111)		38.5 (20)	61.5 (32)				
14	95.1 (77)	4.9 (4)		49.0 (71)	51.0 (74)		50.0 (1)	50.0 (1)				
15+	84.6 (11)	15.4 (2)		53.8 (43)	46.3 (37)		0 (0)	100 (1)				
**Sociodemographic Characteristics**												
**Household poverty**												
Novice nun												
Yes	85.3 (64)	14.7 (11)	1.12 (0.75–1.67)									
No	83.8 (290)	16.2 (56)	1.00									
Household poverty score, mean (SD)				0.97 (0.77)	1.13 (0.83)	0.77 (0.56–1.07)	41.43 (14.55)	42.11 (14.73)	1.00 (0.99–1.00)	4.08 (3.75)	4.80 (3.87)	0.95 (0.91–0.99)
**Current age**												
Continuous												0.99 (0.97–1.01)
**Highest level of education attended**												
None or primary school										31.0 (67)	69.0 (149)	1.00
Secondary school										55.1 (152)	44.9 (124)	2.73 (1.7–4.26)
Post-secondary education										72.0 (77)	28.0 (30)	5.71 (2.83–11.51)

Household poverty was not associated with awareness at menarche in Soroti, Uganda nor in Netrokona, Bangladesh. Similarly, enrolment as a novice nun, indicating participants were likely to come from a poorer household, was not associated with awareness at menarche in Myanmar.

Among adult women working in Mukono, Uganda, household wealth at the time of the survey was associated with a higher level of awareness at menarche, as was a higher level of schooling. Adult women's age was not associated with awareness at menarche, which may suggest no generational difference in awareness.

### Experience at Menarche

We tested the bivariate relationships between awareness of menstruation at menarche and girls' reported feelings at menarche captured in Magway, Myanmar (see Table 3). Girls' who reported being aware of menstruation at menarche had higher odds of reporting they felt happy, prepared, and knew what to do to manage menstruation. However, they also had higher odds of reporting that they felt embarrassed at menarche, and there was no difference in reports of feeling afraid, normal, proud, ashamed. There was also no statistically significant difference in the proportion of girls reporting they felt comfortable to tell their family or female friends.

**Table 3:** Girls' experience at menarche according to their awareness of menstruation in Magway, Myanmar (*n* = 421).

**Experience at menarche**	**Yes, aware % (n)**	**No % (n)**	**OR (95% CI)**
I felt happy	19.5 (69)	6.0 (4)	3.81 (1.74–8.37)
I felt afraid	70.3 (249)	65.7 (44)	1.24 (0.82–1.85)
I felt embarrassed	81.6 (289)	71.6 (48)	1.76 (1.04–2.97)
I felt normal	20.1 (71)	13.4 (9)	1.62 (0.75–3.46)
I felt proud	17.8 (63)	13.4 (9)	1.40 (0.47–4.13)
I knew what was happening	58.5 (207)	37.3 (25)	2.37 (1.34–4.19)
I felt prepared	33.3 (118)	14.9 (10)	2.85 (1.34–6.08)
I felt ashamed	78.0 (276)	73.1 (49)	1.30 (0.90–1.87)
I knew what to do to manage menstruation	83.6 (296)	52.2 (35)	4.67 (3.51–6.29)
It was celebrated in my family/community	6.8 (24)	4.5 (3)	1.55 (0.55–4.36)
I felt comfortable to tell my family about it	50.9 (180)	38.8 (26)	1.63 (0.97–2.74)
I felt comfortable to tell my female friends about it	64.4 (228)	47.8 (32)	1.98 (0.88–4.47)

### Knowledge, Social Support, and Confidence

The relationships between awareness at menarche and menstrual knowledge, comfort with support sources and confidence to manage menstruation are displayed in Table 4. In the two samples implementing a broader menstrual knowledge quiz, awareness at menarche was associated with a test score 5% points higher in Bangladesh and 10% points higher in Myanmar. In Myanmar, girls reported feeling more confident to talk to friends about menstruation and ask a teacher for a pad when they were aware at menarche, and in Bangladesh girls more often reported feeling comfortable to discuss menstruation in general. In Soroti, Uganda, more girls aware of menstruation at menarche reported feeling confident to discuss menstruation with family and friends, but differences were not statistically significant. In Myanmar and Bangladesh, girls aware at menarche reported greater confidence to manage menstruation at school, but not at home. There were no significant differences in confidence managing menstruation in Soroti, Uganda.

**Table 4:** Relationships between awareness of menstruation at menarche and menstrual knowledge, social support, and confidence to manage menstruation.

**Awareness at menarche**	**Myanmar (*****n*** **=** **421)**			**Uganda, Soroti (*****n*** **=** **537)**			**Bangladesh (*****n*** **=** **1,359)**			**Uganda, Mukono (*****n*** **=** **525)**		
	**Yes % (n)**	**No % (n)**	**OR/b (95%CI)**	**Yes % (n)**	**No % (n)**	**OR (95%CI)**	**Yes % (n)**	**No % (n)**	**OR/b (95%CI)**	**Yes % (n)**	**No % (n)**	**aOR^a^ (95%CI)**
**Menstrual knowledge**												
Knowledge test score (M, SD)	58.09 (16.82)	48.58 (18.89)	9.51 (3.99–15.04)				67.52 (23.15)	62.74 (23.44)	4.78 (1.70–7.87)			
**Social support**												
Confident to talk to family member about menstruation	92.4 (327)	83.6 (56)	2.38 (0.94–6.02)	67.7 (159)	61.9 (182)	1.29 (0.98–1.69)				72.3 (188)	63.3 (167)	1.36 (0.98–1.88)
Confident to talk to friends about menstruation (someone at work about menstruation)	79.4 (281)	55.2 (37)	3.12 (1.25–7.78)	60.4 (145)	55.1 (162)	1.24 (0.92–1.69)				53.5 (139)	47.0 (124)	1.15 (0.89–1.40)
Confident to talk to female teacher about menstruation	42.9 (152)	29.9 (20)	1.76 (0.93–3.35)									
Confident to ask a teacher for a pad	36.7 (130)	20.9 (14)	2.20 (1.31–3.68)									
Feel comfortable to talk about menstruation in general							35.6 (165)	22.8 (203)	1.87 (1.38–2.53)			
**Confidence to manage menstruation**												
Confident to manage menstruation at school/work	78.3 (277)	67.2 (45)	1.76 (1.08–2.87)	47.9 (115)	54.4 (161)	0.77 (0.51–1.16)	52.8 (247)	42.3 (377)	1.52 (1.18–1.96)	40.0 (104)	46.2 (122)	0.94 (0.71–1.25)
Very confident to manage menstruation at home				33.8 (81)	38.2 (113)	0.83 (0.52–1.32)	59.2 (277)	56.0 (499)	1.14 (0.84–1.55)	36.5 (95)	42.1 (111)	0.78 (0.60–1.03)

a *With adjustment for poverty index and education level*.

Among adult women in Mukono, Uganda, there were no statistically significant relationships between awareness at menarche and social support or confidence to manage menstruation after adjusting for the significant variation in awareness according to poverty and education level. However, there was a substantial percentage difference with 72% of those aware at menarche comfortable to talk to family about menstruation, compared to 63% of those not aware (*p* = 0.063 after adjustment).

## Discussion

In this study we aimed to describe the proportion of participants across four different study samples aware of menstruation at menarche, test variations in self-reported awareness according to sociodemographic characteristics and age at menarche, and to test the associations between awareness at menarche and menstrual knowledge, social support, experience at menarche and confidence to manage menstruation. Awareness of menstruation at menarche varied across the included samples. Among our adolescent samples, awareness was not associated with household resources, but was positively associated with older age at menarche. Experiences of menarche were only assessed in Myanmar, and we found that awareness of menstruation was associated with feeling happy, prepared, and knowing what to do to manage menstruation. However, awareness was also associated with more frequently reported feelings of embarrassment at menarche, and not associated with differences in reported fear or shame. We found that awareness at menarche may also signal greater knowledge of menstruation more broadly, with awareness associated with improved scores on a menstrual knowledge test in the two samples including these (Myanmar and Bangladesh) (29, 34). In Myanmar and Bangladesh samples, awareness at menarche was positively associated with confidence or comfort to discuss menstruation with some support sources, and confidence to manage menstruation at school. Taken together, our findings add to the descriptive value of awareness of menstruation at menarche as an indicator for menstrual health. Further, findings suggest that improving levels of awareness may help girls have a more positive experience of menarche, but that positive education is needed to dispel higher levels of embarrassment reported.

Awareness at menarche ranged from 34% of girls in Bangladesh, to 84% of respondents in Myanmar. This variability is consistent with that observed across studies reporting on this measure to date (11, 23, 24). Awareness at menarche in Netrokona, Bangladesh (34%), was similar to that reported by Alam et al. in their national sample (36%) and the Bangladesh National Hygiene Survey (32%) (11). In our adolescent samples we did not find a relationship between awareness at menarche and household wealth, although note that studies were undertaken in restricted samples. As studies were undertaken in single geographic settings we did not have scope to compare rural and urban residence to contrast findings to those reported by the JMP (11). In our sample of adult women working in Mukono, Uganda we found that higher levels of education and lower current poverty were associated with greater levels of awareness at menarche. The significant time lapse between menarche and women's current reports of household resources makes this association more difficult to interpret. It is unclear if current socioeconomic status reflects women's access to resources in adolescence (at the time of menarche) or education, or if an unmeasured confounder, such as parental education, is driving this effect. However, differences remain important to identify and adjust for in subsequent analyses as women's experience of menstruation is also likely to vary with poverty and education level. We did not find a generational difference in awareness at menarche across our sample of adult women (18–45) Mukono, Uganda.

Consistent with analysis by the JMP of national data from Egypt and Bangladesh, age at menarche was positively associated with awareness in Bangladesh and Myanmar, and a similar pattern emerged among schoolgirls in Uganda although was not statistically significant. Girls experiencing menstruation later have greater opportunity to have learned about menstruation from school education, parents, or peers prior to menarche, than those experiencing menarche earlier. Early menarche has been identified as a risk factor for a range of deleterious mental health and sexual and reproductive health outcomes in high- middle- and low-income contexts (39–41). Earlier fertility, sexual attention and substance use have been proposed to mediate this relationship in high-income country studies (39, 42), and early marriage has been highlighted as influencing these relationships in some low- and middle-income country settings (41). Future studies should investigate experiences of menstruation and reproductive health knowledge as other potential mediators of this relationship.

Among schoolgirls in Myanmar, awareness of menstruation at menarche was associated with some more positive emotions at menarche, including happiness and feeling prepared. This differed from JMP analysis of national data from Egypt which found no difference in knowing what to do at menarche between those who were and weren't aware of menstruation (11, 27). Counter to data from Bangladesh and Egypt, awareness of menarche in our sample in Myanmar was not associated with fear at menarche. In our sample, more girls who were aware of menstruation reported feeling embarrassed at menarche, which may be consistent with findings from Bangladesh that more girls aware of menstruation reported feeling shame (11). This could suggest that the early information girls receive about menstruation already reinforces its characterization as something shameful and embarrassing (19, 43–45). Accurate and positive information about the menstrual cycle is needed to support menstrual health and improve girls' experience at menarche (44, 46).

In the two samples (Myanmar and Bangladesh) assessing knowledge about the biology of menstruation and its links to reproduction, we found that awareness of menstruation at menarche was positively associated with scores on a knowledge test. This is consistent with the hypothesis that girls aware of menstruation at menarche may have also received other information about menstrual biology, provided by family members or through school education programs. This suggests that although measuring awareness at menarche only captures the very minimal level of knowledge about menstruation, the indicator may also serve to suggest that some other information has been shared.

We found that awareness of menstruation at menarche was associated with feeling confident to talk about menstruation with support sources in Myanmar and Bangladesh, with a similar association observed among schoolgirls in Soroti, Uganda but not statistically significant. There may be multiple reasons for this association. If friends or parents have shared menstrual information with girls, they may then feel comfortable continuing to seek support from those individuals. Where menstrual education has been provided in schools, this may support girls to feel more comfortable seeking advice or support from teachers. Girls' confidence to manage menstruation at school was positively associated with awareness at menarche in Myanmar and Bangladesh. This may suggest that early exposure to information and preparation for menarche help to build confidence in managing menstruation. However, alternative explanations exist. Girls receiving information in time for menarche may also receive greater family support in general, support from peers, or attend school in a more supportive environment for managing menstruation. Notably in a multivariable comparison of predictors of confidence to manage menstruation in the sample of schoolgirls in Bangladesh included in this study, awareness at menarche was not significantly associated with confidence after adjustment for other social and environmental factors (33). Our analysis was not designed to test causal links between awareness or experiences at menarche and girls' social support or confidence, and longitudinal designs are needed. The bivariate associations investigated are relevant for interpreting awareness at menarche as an indicator at national levels, suggesting this measurement can provide inferences about early menstrual knowledge and confidence. Future studies should investigate the role of early menstrual education, along with the sources of information and quality of education received to better understand these relationships.

We did not find significant associations between awareness at menarche and adult women's current experiences of menstruation after adjusting for sociodemographic differences in awareness. This may be expected given the significant time delay between menarche and our adult sample. While not statistically significant, more women aware of menstruation at menarche reported currently feeling comfortable discussing menstruation with family members which could suggest that a more open early environment continues to influence comfort discussing menstruation over time.

## Strengths and Limitations

Our investigation leveraged four existing studies undertaken to serve different research questions. This provided a diverse set of samples to report the prevalence and test associations with awareness at menarche adding strength to the findings, however none of the included studies were designed to investigate this research question. Studies were not selected systematically; they were all research efforts in which the first author had been involved and had access to the primary study data. The reliability of self-reported awareness of menarche has not been tested, although one past study investigating women's reported age at menarche has suggested stability in self-report over time (47). If measures to assess awareness at menarche are taken up across contexts, piloting will be required to ensure the questions used are acceptable and well-understood by respondents in varied settings. As noted above, questions used assessed only awareness at menarche, and not the quality or source of early information which may be important to understand in informing responsive interventions. All studies in our investigation reported retrospective cross-sectional data. Longitudinal studies would be best placed to test directional relationships between awareness at menarche and girls' subsequent menstrual experiences. Our analyses were designed to test correlates of awareness as an indicator. Across all four studies, we only assessed participants' access to social support for menstruation through their self-reported comfort or confidence to discuss menstruation with support sources. This does not capture whether support was sought or provided to participants. More nuanced assessment of support received for menstruation, and the type of information discussed by support sources, should be explored in future studies.

## Conclusions

By drawing together relevant data from four different research efforts, we have investigated the variability of awareness of menstruation at menarche and tested the hypothesis that this indicator may also suggest greater access to social support, menstrual knowledge, and early confidence in managing menstruation. Further, our findings add to the qualitative evidence that knowing about menstruation prior to menarche may mean girls feel more prepared and positively at menarche, however our quantitative findings suggest that alone this knowledge does not dispel embarrassment or fear. More information and support are needed to dismantle the stigma surrounding menstruation, and complimentary indicators will be needed to comprehensively monitor progress toward menstrual health at global, national, or subnational levels. Future studies may use the results presented here to inform targeted investigations of the role of early information and menstrual health literacy on experiences of menstruation in adolescence and over the life-course.

## Data Availability Statement

The data analyzed in this study is subject to the following licenses/restrictions: Data from studies in Magway, Myanmar, and Soroti and Mukono, Uganda are available upon reasonable request from JH. Study data from Netrokona, Bangladesh may be requested from LS. Requests to access these datasets should be directed to JH, julie.hennegan@burnet.edu.au and LS, lidwiensol@gmail.com.

## Ethics Statement

Each study received ethical approval consistent with its location and study team: Magway, Myanmar: The study was approved by the Department of Medical Research Ethics Review Committee (035/16) in Myanmar and the Alfred Human Ethics Committee (59/16) in Australia. All participants provided written assent to participate and had written parental consent for participation. Soroti, Uganda: Ethical approval was provided by Johns Hopkins School of Public Health Institutional Review Board (IRB approval no: 00009073) and the Mildmay Uganda Research Ethics Committee (MUREC) (approval ref: 0212–2018). The Uganda National Council for Science and Technology (UNCST) approved the study (ref: SS279ES). Girls provided written informed assent or consent to participate. Parents were informed about the study through information sheets, parent-teacher meetings at each school and through contact with teachers. Netrokona, Bangladesh: This study was approved by the Erasmus Research Institute of Management (IRB 2016-09), Erasmus University Rotterdam, and the Directorate of General Secondary Education in Bangladesh. The District Education Officer also granted approval for data collection and girls provided written informed assent. Written informed consent from the participants' legal guardian/next of kin was not required to participate in this study in accordance with the national legislation and the institutional requirements. Mukono, Uganda: Ethical approval was provided by Makerere University School of Public Health Higher Degrees, Research and Ethics Committee (HDREC: 739) and Johns Hopkins Bloomberg School of Public Health Institutional Review Board (IRB: 00010015). The Uganda National Council for Science and Technology (UNCST) approved the study (ref: SS 5143). All women provided written informed consent.

## Author Contributions

JH conceived this investigation, conducted analysis, and drafted the manuscript. Study design, data collection, and curation were led by JH, CS, and KS for Soroti, Uganda, LS and HA for Netrokona, Bangladesh, and by JH, JB, SK, FM, and KS for Mukono, Uganda. For Magway, Myanmar, ZS, KT, and PA formed part of the core investigator team leading study design, data collection and curation. All authors critically reviewed the drafted manuscript and have approved submission of the final manuscript.

## Funding

This investigation was funded by an NHMRC Ideas Grant (GNT2004222). PA was supported by a NHMRC Early Career Fellowship (#1145228). We gratefully acknowledge the contribution to this work of the Victorian Operational Infrastructure Support Program received by the Burnet Institute. The study in Magway, Myanmar was funded through the Australian NGO Cooperation Program funded by the Australian Department of Foreign Affairs and Trade, and Water AID Australia. Research in Soroti, Uganda was supported by the Case for Her and the Osprey Foundation of Maryland. The Ritu trial (Netrokona, Bangladesh) was funded by the Embassy of the Kingdom of the Netherlands in Dhaka, Bangladesh with additional support from Simavi, the Netherlands. The study in Mukono, Uganda was supported by the Osprey Foundation of Maryland.

## Conflict of Interest

The authors declare that the research was conducted in the absence of any commercial or financial relationships that could be construed as a potential conflict of interest.

## Publisher's Note

All claims expressed in this article are solely those of the authors and do not necessarily represent those of their affiliated organizations, or those of the publisher, the editors and the reviewers. Any product that may be evaluated in this article, or claim that may be made by its manufacturer, is not guaranteed or endorsed by the publisher.
